# Severe Suicidality in Athletes with Chronic Traumatic Encephalopathy: A Case Series and Overview on Putative Ethiopathogenetic Mechanisms

**DOI:** 10.3390/ijerph18030876

**Published:** 2021-01-20

**Authors:** Alessandra Costanza, Michalina Radomska, Francesco Zenga, Andrea Amerio, Andrea Aguglia, Gianluca Serafini, Mario Amore, Isabella Berardelli, Yasutaka Ojio, Khoa D. Nguyen

**Affiliations:** 1 Department of Psychiatry, Faculty of Medicine, University of Geneva (UNIGE), 1211 Geneva, Switzerland; 2 Department of Psychiatry, ASO Santi Antonio e Biagio e Cesare Arrigo Hospital, 15121 Alessandria, Italy; 3 Faculty of Psychology, University of Geneva (UNIGE), 1206 Geneva, Switzerland; michalina.radomska@outlook.com; 4 Department of Neurosurgery, City of Health and Science Hospital, 10126 Torino, Italy; zengafra@hotmail.com; 5 Department of Neuroscience, Rehabilitation, Ophthalmology, Genetics, Maternal and Child Health, Section of Psychiatry, University of Genoa, 16132 Genoa, Italy; andrea.amerio@unige.it (A.A.); andrea.aguglia@unige.it (A.A.); gianluca.serafini@unige.it (G.S.); mario.amore@unige.it (M.A.); 6 IRCCS Ospedale Policlinico San Martino, 16132 Genoa, Italy; 7 Department of Psychiatry, Tufts University, Boston, MA 02111, USA; 8 Suicide Prevention Center, Department of Neurosciences, Mental Health and Sensory Organs, Sant’Andrea Hospital, Sapienza University of Rome, 00189 Rome, Italy; isabella.beradelli@uniroma1.it; 9 National Center of Neurology and Psychiatry, Department of Community Mental Health Law, National Institute of Mental Health, Tokyo 187-8553, Japan; ojiotokio@gmail.com; 10 Department of Microbiology and Immunology, Stanford University, Palo Alto, CA 94304, USA; khoa.d.nguyen@gmail.com; 11 Tranquis Therapeutics, Palo Alto, CA 94304, USA; 12 Hong Kong University of Science and Technology, Hong Kong, China

**Keywords:** chronic traumatic encephalopathy, contact sports, neuropsychiatric symptoms, suicide, suicidal ideation, suicide attempt, suicidal behaviour

## Abstract

Chronic traumatic encephalopathy (CTE) results from repetitive brain injuries and is a common neurotraumatic sequela in contact sports. CTE is often accompanied by neuropsychiatric symptoms, which could escalate to suicidal ideation (SI) and suicidal behaviour (SB). Nevertheless, fairly limited emphasis about the association between suicidality and CTE exists in medical literature. Here, we report two cases of retired professional athletes in high contact sports (boxing and ice hockey) who have developed similar clinical trajectories characterized by progressive neuropsychiatric symptoms compatible with a CTE diagnosis and subsequent SB in its severe forms (medical serious suicide attempt (SA) and completed suicide). In addition to the description of outlining clinical, neuropsychological, neuroimaging, and differential diagnosis elements related to these cases, we also hypothesized some mechanisms that might augment the suicide risk in CTE. They include those related to neurobiological (neuroanatomic/neuroinflammatory) dysfunctions as well as those pertaining to psychiatry and psychosocial maladaptation to neurotraumas and retirement from professional competitive activity. Findings described here can provide clinical pictures to improve the identification of patients with CTE and also potential mechanistic insights to refine the knowledge of eventual severe SB development, which might enable its earlier prevention.

## 1. Introduction

Contact sports athletes are commonly exposed to concussions [1]. After the first occurrence, susceptibility to subsequent concussions is progressively augmented in both frequency and severity, compounding brain injuries [2]. In combative sports such as boxing and mixed martial arts concussions are sustained by rotational forces [3,4], whereas in ice hockey multidirectional acceleration and deceleration contribute to this type of neurological injury. Notably, the incidence of concussion increases in contact sports athletes at the professional level and is still very much underestimated [5,6]. Acute and long-term neuropsychiatric consequences associated with injuries of contact sports athletes were first documented in boxing, with the early definition of “punch-drunk syndrome” [7] and “dementia pugilistica” [8] in the 1920–30s. On the contrary, in ice hockey, another contact sport that was presented in the medical literature as a mixture of “finesse and controlled aggression” [9] and in poetry as a “combination of ballet and murder” [10], reports are much more recent with special attention on acute brain injuries rather than chronic psychiatric sequelae [5,6]. In subsequent decades, a more comprehensive spectrum of neurological anomalies in contact sports was classified, ranging from acute “knockout” and “amnesia” to “groggy state” (particularly in boxing) and, most recently, to long-term chronic progressive traumatic encephalopathy (CTE). 

CTE was first defined as progressive deterioration of cognitive function that is induced by consistent exposure to traumatic brain injuries [11,12,13]. Clinical symptoms of CTE include memory loss, behavioural and personality alterations, as well as parkinsonism and speech/gait abnormalities, all of which are accompanied by atrophy of multiple cortical brain regions [13,14]. Along with chronic traumatic brain injury, CTE has been an evolving clinical concept [15,16,17] that highlights the crucial involvement of repetitive head traumas in the development of neuropsychiatric symptoms in contact sports professionals [18]. In this regard, the characteristic triad of cognitive decline, failing in anger management, and depression has been the defining neuropsychiatric manifestations of CTE [19]. These manifestations are highly evident in some patient backgrounds such as in athletes, military men, and victims of certain crimes who are consistently exposed to neurotraumatic events [20]. While CTE can be suspected by the clinical status of the patient, a histopathological documentation of tauopathies of the patient’s brain, which are correlated with neuropsychiatry impairments, is the only definitive procedure to confirm the diagnosis of CTE [21]. 

The link between contact sports and CTE has been a growing concern for youth athletic associations [22,23] and professional sports leagues [24,25] in recent years, and there is speculation that some of these injuries could develop into SB later in life [26,27,28]. Progressive neuropsychiatric symptoms resulting from CTE could lead to suicidal ideation (SI) and eventually suicidal behaviour (SB), especially the more severe forms of SB such as medical serious suicide attempt (SA) and completed suicide (for recent literature reviews, see [29,30]). For example, CTE was confirmed in post-mortem examinations of over 100 former National Football League players and it was hypothesized that suicide in four of them could have resulted from CTE-induced behavioural changes, but it is difficult to make a direct connection in these cases [31]. 

Similar to the scarcity of data on SI and SB in patients with comorbid illness [32,33,34], fairly little is known about the association between CTE and suicidality to date. Thus, the primary objective of this report is to describe the occurrence of severe suicidality in two retired contact sports professionals who exhibited CTE. Additionally, we discuss some potential mechanisms that might augment the risk for suicide in CTE, including those related to neurobiological dysfunction as well as those pertaining to psychiatry and psychosocial maladaptation to neurotraumas. Both presented patients had given and signed their consent to describe and publish, in anonymized form, their medical story. 

## 2. Case Studies

### 2.1. Case 1

The first case was an 83-year-old Caucasian patient who started boxing at the national level at the age of 17. He became well recognized as “the silent prince of boxing”, because of a personality described as introverted and determined. His career lasted for about 10 years until it was terminated by a wrist fracture during training for a crucial match. In 1998, approximately 40 years after sports retirement, the patient suffered the first major depression episode that culminated in a SA by severe drug overdose which was characterized by high lethality and intentionality as evidenced by the clinical report (about 14 mg paracetamol at an estimated 4 h after ingestion equal to 4815.3 mcg/mL, reference values: 10–20; levothyroxine; and a high blood alcohol level of 3.3 g/L). The subject was found in his cellar for the purported purpose of concealing his actions. He was discovered by his wife in a state of unconsciousness and immediately sent to an emergency department where he registered a 4 on the Glasgow Coma Scale. The severity of intoxication and its somatic consequences required intensive care hospitalization. The patient was diagnosed with a major depression episode and treated with tricyclic antidepressants (clomipramine) with clinical benefit on the depressive symptoms. A brain CT scan showed diffuse moderate cortical and subcortical atrophy. An indication for initiating anticholinesterase drugs was not considered on the basis of these alterations. From a clinical point, a formal diagnosis of initial mild cognitive impairment (MCI) or dementia was not retained. However, on this occasion, no neuropsychological evaluation was made nor were further diagnostic investigations performed.

**Figure 1:**
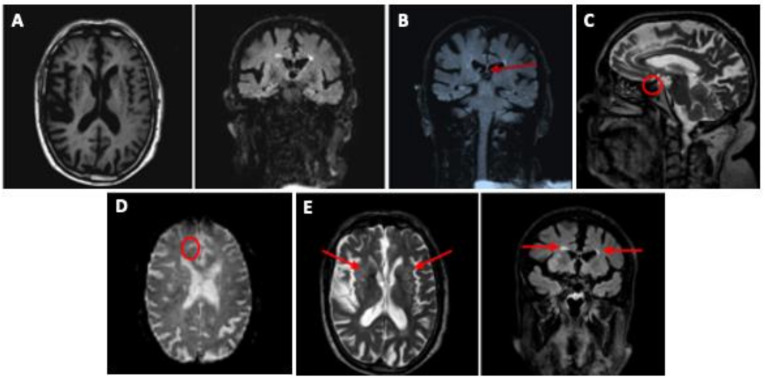
Brain MRI of the boxer. **A.** Axial T1 (left) and coronal flair (right) sections showing significant fronto-temporal-parietal cortical and sub-cortical atrophy with ventricular dilatation, and mild hippocampal atrophy bilaterally. **B.** Coronal flair section showing slight cavum septum pellucidum. **C.** Sagittal T2 section showing slightly atrophic pituitary gland. **D.** Axial proton density weighing (DP) section showing diffuse axonal injury. **E.** Axial T2 (left) and coronal flair (right): numerous vascular lacunae in the basal ganglia bilaterally (cribrate aspect) and per-ventricular leukoaraiosis.

In October 2007, his first neuropsychological assessment (Table 1) mainly revealed moderate memory deficits and impairment of executive functioning. A treatment of galantamine (24 mg/day) was subsequently introduced but his neuropsychiatric condition rapidly deteriorated. In April 2008, a brain MRI showed a more precise localization of the cortical and sub-cortical atrophy with bilateral fronto-temporo-parietal predominance as well as hippocampal involvement (Figure 1A), a mild cavum septum pellucidum (Figure 1B), slightly atrophic pituitary gland (Figure 1C), diffuse axonal injuries (Figure 1D), and numerous millimetric vascular lacunae and supratentorial leukoaraiosis in the basal ganglia, particularly around the ventricles (Figure 1E). Based on these findings, his differential diagnosis suggested the presence of CTE, mixed dementia of degenerative and vascular origin, and Alzheimer’s disease (AD). The diagnosis of fronto-temporal dementia with behavioural variant (FTD-bv) received less attention because of the presence of the hippocampal pathology and the fact that the most severe behavioural disturbance appeared later (see below) than cognitive decline.

In August 2008, a second neuropsychological assessment (Table 1), confirmed the severe impairment in episodic memory and executive functions while no extrapyramidal signs were detected. A Clinical Dementia Rating (CDR) score of three was retained. His medication (galantamine) was replaced by memantine (10 mg/day) and psychotropic drugs (alprazolam 0.50 g/day and trazodone 150 mg/day). In September 2008, the patient was referred to the psycho-geriatric department at our institution due to depressive symptoms, increased verbal and physical aggressiveness, and sleep disturbances. Antipsychotic (haloperidol 3 mg/day) was halted because of the rapid occurrence of parkinsonism and replaced by a combined treatment of citalopram (10 mg/day), quetiapine extended-release formulation (300 mg/day), and gabapentin (900 mg/day). This regimen rapidly lost its initial effectiveness in curbing neuropsychiatric and cognitive impairment quickly over the next three months. Specifically, his communication abilities were markedly reduced, and the patient also suffered from visual hallucinations as he was found boxing against imaginary adversaries. 

A second severe SA was made in January 2009 by hanging. The subject was once again discovered by his wife when she went looking for him after leaving their home unannounced. In this instance the rope broke and there were no severe somatic injuries with the exception of minor orthopaedic sequelae from falling to the ground. As a result of the second attempt, lithium and clozapine therapies were considered but were not administered due to moderate renal insufficiency and the occurrence of leukopenia, respectively. Notably, the patient never verbalized SI. He was admitted to the county psychiatric hospital also because of additional psychomotor agitation and significant sleep disturbances, all of which were markedly alleviated by the introduction of clomethiazole (500 mg/day) and quetiapine immediate-release formulation (100 mg/day). The patient was then discharged and continued to be cared for by his wife until his death due to natural causes.

### 2.2. Case 2

**Figure 2:**
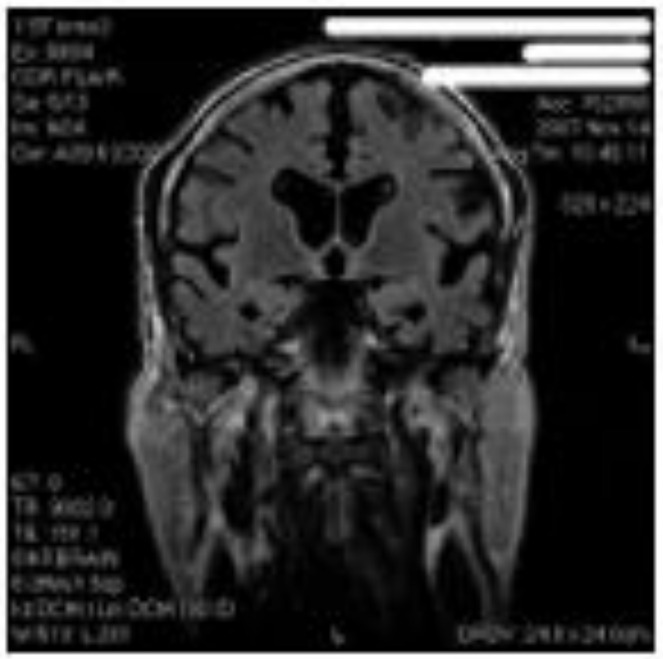
Brain MRI of a 74-year-old former ice hockey player. Coronal T1 showing significant fronto-temporal-parietal cortical and sub-cortical atrophy with ventricular dilatation, hippocampal atrophy bilaterally, and diffuse axonal injury.

The second case was a 74-year-old Caucasian who started practicing hockey at the age of 16 and played at the national level as “the best hockey player of the epoch” or “the king of forwards”, the descriptions given to him by the press due to his determination and constant firmness “in attacking as well in character”. He suffered at least two medically documented concussions during his career (at that time, hockey players did not have protective head gear). In 2006, about 35 years after his retirement from sports for age restriction, the patient exhibited increasing difficulties in managing daily habits and deficits in memory and attention/concentration. In November 2007, a neuropsychological assessment (Table 1) revealed severe impairments in anterograde, retrograde, and visuospatial memories, visuospatial orientation, and executive functions. memories and executive functions. An encephalic MRI showed a marked diffuse cortical and sub-cortical atrophy with bilateral hippocampal involvement, and axonal injuries (Figure 2). The same differential diagnoses reported for the previous patient were also discussed in this case. Donepezil (10 mg/day) was introduced.

At the end of July 2008, the patient was admitted to our psycho-geriatric department because of depressive symptoms, soliloquies, and nocturnal hallucination, consisting in unknown adversaries menacing and hitting him. This florid symptomatology was preceded by emotional lability, with unusual episodes of uncontrolled impatience, impulsivity, irritability, unmotivated hilarity, and childishness. Citalopram (20 mg/day), haloperidol (3 mg/day), lorazepam (2–2.5 mg/day), and valproate (600 mg/day) were introduced. His neuropsychiatric symptoms became aggravated over a period of 4–5 months with the additional appearance of severe language deficits and a complete loss of autonomy. His nocturnal visual hallucinations also dramatically worsened. Increasing dose of haloperidol to 5 mg/day and its subsequent replacement with risperidone (2 mg/day) triggered a sudden and important extra-pyramidal syndrome, which required a psychiatric hospitalization in January 2009. The introduction of quetiapine (600 mg/day), mirtazapine (30 mg/day), and trazodone (50 mg/day) alleviated psychiatric symptoms and signs of parkinsonism. Memantine (10 mg/day) was initiated in substitution of donepezil. Nevertheless, the patient’s condition was never normalized, and in 2009 he evaded caregivers and completed suicide from the seventh floor of his building by self-defenestration. In this case the intentionality and lethality of the act can be considered severe. As with Case 1, the patient never verbalized SI.

## 3. Discussion

Only within the last 10 years has the potential for suicidality that is associated with earlier head trauma and CTE been gaining prominence in the literature. On the strength of the accumulating evidence, as indicated by McCrory et al. [35], a number of recommendations have been stated. Many of these concern prevention (e.g., with helmets and other head protection devices), recognition of somatic consequences even if prodromal, and clinical evaluation [35]. Importantly, these clinical measures include an increased concern for the possible onset of neuropsychiatric symptoms, including suicidality. An initial somatic assessment in individuals exposed to concussions related to sports involves requesting a neuropsychological and psychiatric evaluation. To our knowledge, there are no detailed guidelines regarding psychiatric evaluation. However, in practice, the latter makes use of: (a) an in-depth interview with a psychiatrist that focuses on depressive symptoms and suicidal ideation with open-ended questions; (b) administration of scales such as the Montgomery–Åsberg Depression Rating Scale (MADRS) [36] and Beck Depression Inventory-II Scale (BDI-II) [37] for determining the absence/presence and severity of depression (specifically, item 9 of the BDI-II concerns suicidality, with a score describing its presence and severity); (c) if this item is positive or concerns are major, administration of specific scales for suicidality, such as the Beck Scale for Suicide Ideation (SSI) or, more recently, the Columbia Suicide Severity Rating Scale (C-SSRS) that is both a suicidal ideation and behaviour rating scale (in particular, it rates an individual’s degree of suicidal ideation on a scale ranging from “wish to be dead” to “active suicidal ideation with specific plan and intent and behaviors”) [38]; (d) an in-depth interview with the family to assess risk/protection factors and the possible nature of family support that may not have emerged in the interview with the patient; (e) evaluation of the indication for a pharmacological therapy for the presence of depression and possibly for SI (in addition to evaluating lithium and clozapine, olanzapine may reduce impulsivity and help to alleviate, as described by many patients, their deep "moral pain" related to suicidal ideation [39]; and (f) possible hospitalization in a psychiatric setting. Although more directly related to depression rather than suicidality, the Baron Depression Screener for Athletes (BDSA) is a brief and reliable assessment tool that was developed in the US and validated in Japan and can be used to help identify and prevent the escalation of suicidality [40].

To the best of our knowledge, cases of boxing-related CTE have been documented [7,18], but reports of ice hockey-related CTE are absent in the literature. Nevertheless, the evolution of the two described cases have some similar clinical presentations that are compatible with a possible diagnosis of CTE and AD. Both patients were exposed to repeated concussions without any form of cranium protection, which now are mandatory [35] and exhibited cortical and sub-cortical atrophies with hippocampal pathology in their cerebral imaging. The occurrence of a “groggy state”, characterized by personality changes [18], was well recognizable in both patients. In the boxer, these symptoms manifested with progressive traits of rigidity, social isolation, and sadness, given his introverted personality. The mood shift was more apparent for the hockey player with emotional lability, accompanied by substance abuse and harsh conjugal conflicts. Both patients also exhibited subsequent major depressive episodes and psychotic symptoms upon their admittance to the psycho-geriatric department with notable visual hallucinations, which involved imaginary adversaries menacing and hitting the patients. With regard to cognition, declines began several decades following their completion of professional sports (50 and 35 years for the boxer and ice hockey player, respectively). In the case of the boxer, the latency was longer than the periods described in published reports and might result from the particular brevity of his professional career (7–10 years vs. 20 years in the case of the ice hockey player). Cognitive impairments in both patients were also related to memory and attention/concentration deficits, which eventually aggravated to disturbances in speech and interpersonal communication (anomia, motor and sensitive aphasia, frequent neologisms, and echolalia). Interestingly, before the introduction of the antipsychotic medication, we did not observe any signs of parkinsonism in either patient, which may occur in CTE but is not as well defined in patients with Parkinson’s disease [41]. The patients, however, exhibited very pronounced sensitivity to antipsychotics, especially haloperidol and risperidone, which triggered immediate extrapyramidal signs. In both patients, this medication-induced parkinsonism assumed the form of a rigid-akinetic syndrome with severe gait disturbances and disequilibrium in the absence of tremor. While extrapyramidal side effects are rare in patients with probable AD diagnosis, we postulate that this earlier and heavier iatrogenic parkinsonism in these two cases might result from substantia nigra degeneration. In addition, the notable gait and equilibrium disturbances could be contributed to damage of afferent and efferent connections in the cerebellum in the absence of cerebellar ataxia. Alternatively, hypersensitivity to antipsychotic medications that results in parkinsonism might also suggest probable FTD-bv. Nevertheless, this diagnosis was not primarily retained in both cases because of the presence of hippocampal pathology.

While these collective clinical features are consistent with neuropsychiatric manifestations in CTE [15,16,18], they are subject to some important limitations. First of all, the diagnostic criteria for CTE is still constantly evolving and therefore there are unknowns associated with its neuropathology. Secondly, retrospective quantification of the frequency of concussion in these two patients was not feasible. Thirdly, the “pre-morbid” state of the patients overshadowed a precise and objective reconstruction of their previous personalities as data about their traits are mostly obtained by anecdotal stories from relatives. Lastly, due to the absence of post-mortem examinations, differential diagnosis between AD or FTD-bv and CTE remained very arduous as these neurological conditions could only be accurately defined by histological examination. It is worth noting that difficulties in distinguishing CTE from AD do exist as corroborating epidemiological studies have shown that head injuries increase the risk of MCI and AD [42,43]. For instance, risk for MCI and early onset AD increased by 5-fold in former athletes who suffered multiple concussions in comparison to those of retirees without a history of concussion and of the general population [44]. In these two cases, possible AD diagnosis was attributed to both patients as their neuropsychiatric symptoms were well-correlated with progressive neurodegeneration in the brain. The presence of SB in these two cases was also consistent with the increased risk of suicidality in patients with AD [45,46,47,48,49]. However, unlike AD, in both cases the cognitive and psychiatric aggravation was rapid (within 2–3 years) and consistent with previously documented progression of these symptoms in CTE [50]. 

A commonality between the cases is the severity of SB. To achieve a consensus definition and operational criteria for a "medical serious" SA (MSSA), a review of more than 60 papers addressing various types of SA was undertaken. Based on this research an integrative and comprehensive set of criteria was developed for defining the severity of SA that comprised three key dimensions: medical lethality, potential lethality of the method used, and severity of the objective circumstances of the suicide intent. It is strongly encouraged that clinicians and researchers are aware of the attendant components when using the terms severe SA or MMSA [51].

Despite diagnostic caveats and nuanced differences in the clinical presentations of the cases, the history of repeated traumatic head injuries is the unifying suspected etiology of their neuropsychiatric condition. More importantly, these symptoms are resistant to pharmacological intervention as such treatments failed to prevent SI and SB. Several factors might contribute to the SI and SB in these patients, including deregulation of neurochemical pathways under the impact of head traumas as well as other psychosocial factors, which is discussed in detail below.

### 3.1. Neurobiological Hypotheses for the Development of Suicidality in CTE

With regard to biological abnormalities in CTE, it has been shown that a neurometabolic signalling cascade is acutely triggered by traumatic brain injuries before the chronic encephalopathy takes place. This event is characterized by an imbalance of sodium and potassium due to hypersecretion of neurotransmitters as well as an increase in calcium within the axons. These electrolytic changes result in activation of calpain protease and subsequent destruction of neuronal cytoskeleton structures, and ultimately in irreversible axonal damage [52]. Such structural alterations are also marked by elevation in tau [53,54,55] and neurofilament light polypeptide (NFL), normally located in unmyelinated cortical axons and deep nervous tissue layers, respectively, in the cerebrospinal fluid after traumatic brain insults. Indeed, clinical evidence from boxers has revealed that these biomarkers, especially total tau proteins, are elevated within a week after their match, suggesting the presence of CTE-associated axonal damage in these athletes [56].

**Figure 3:**
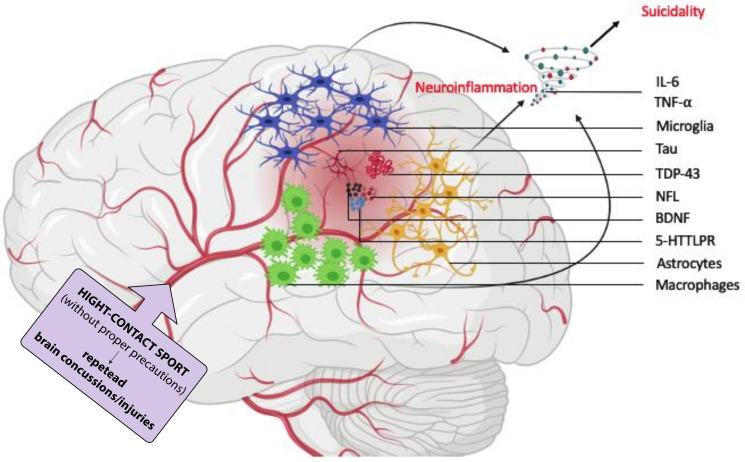
Putative neurobiological mechanism of suicidality development in chronic traumatic encephalopathy (CTE). Chronic progressive head traumas cause axonal damage resulting in pathogenic expression of tau and TAR DNA binding protein 43 (TDP43) proteins and induction of other factors such as neurofilament light polypeptide (NFL), brain-derived neurotrophic factor (BDNF), and serotonin transporter 5-HTTLPR. Consequently, some of these factors trigger immunoreactivity from infiltrated macrophages as well as brain resident microglia and astrocytes. The production of neuroinflammatory cytokines such as interleukin 6 (IL-6) and tumour necrosis factor alpha (TNF-α) by these cells sustain a neuroinflammatory cascade which potentiates—in synergy with BDNF and 5-HTTLPR—the risk for suicidal ideation and behaviour in CTE.

Moreover, such microscopic injuries in the central nervous system can trigger neuroinflammatory processes that are orchestrated by microglia, astrocytes, and macrophages (in the context of compromised blood brain barrier integrity) [57] (Figure 3). Upon recognition of these abnormally expressed protein aggregates, these cells elicit a robust inflammatory reaction, characterized by the release of cytokines and chemokines to amplify and sustain this response. Such inflammatory milieu might potentiate the risk for SI and SB as they have been convincingly shown to be positive correlates of suicidality [58,59]. In this regard, patients with SA exhibit high levels of several inflammatory markers, such as IL-6 and TNF-α [60,61], all of which were found to be elevated in the cerebrospinal fluid of subjects with traumatic brain injuries [57,62]. 

Other neurobiochemical changes, which have been documented in patients at risk for SI and SB, such as serotonin transporter (5-HTTLPR) and brain-derived neurotrophic factor (BDNF) [63,64,65,66,67], are also found to be altered in subjects with traumatic brain injuries [68,69]. The possible involvement of these neurological pathways in the host response and/or maladaptation to traumatic brain injuries and CTE development still remains to be elucidated.

CTE has also been suggested to be associated with dementia as initial studies linked this condition to genetic polymorphisms in several dementia-associated genes, such as apolipoprotein E allele 4 (APOE-ε4) and the β-amyloid-degrading enzyme neprilysin [70,71]. Aside from tau [72], other protein inclusions that are associated with various forms of AD and dementia, such as TAR DNA binding protein 43 (TDP-43) [73] and neurofibrillary tangles [53,74], are also detected in subjects with CTE [14]. Collectively, these findings suggest the possible existence of a neurobiological pathway that is affected in both CTE and dementia [75,76]. Since dementia has been associated with increased suicide risk [45,46,77], it is likely that suicidality might be the ultimate consequence of this common neuropathology in CTE and dementia. Specifically, these patients may experience pronounced social stigma for this inevitable degeneration in cognitive status, eventually increasing their risk for SI and SB. Nevertheless, we want to stress the hypothetical nature of the association between these signal pathways, dementia and suicidality, and that confirmatory studies are warranted to further investigate possible mechanistic linkages amongst this triad of neurological anomalies.

### 3.2. Psychiatric and Psychosocial Hypotheses for Suicidality Etiology in CTE 

While the involvement of dementia-related cognitive decline in CTE-associated suicidality is still a subject of debate, several other neuropsychiatric and psychosocial conditions have been proposed to be the underlying primers that potentiate suicide risk in subjects with CTE. For instance, the occurrence of major depression, a constitutive sign of CTE, represents one of the strongest risk factors for SI and SB [11,12,13]. Hopelessness is also known as a pivotal factor that may eventually precipitate to SI and SB, which appeared to be an independent and more powerful risk factor of suicidality compared to depression [78]. In this regard, it is plausible that both patients’ neuropsychiatric and psychosocial conditions, a sort of drift in two athletes who had been famous and idolized, trigger a sense of hopelessness. Alternatively, the unpredictable nature of CTE progression might also aggravate this feeling [45,46]. 

Another critical aspect in the pathway toward suicidality can be represented by two constructs that are deeply investigated in patients with somatic diseases, the feeling of absence of Meaning in Life (MiL) and demoralization [79,80]. After its introduction into the psychiatric literature [81], MiL was recently described as one’s perception of their own coherence, purpose, and significance [82] while demoralization was defined “a persistent failure to cope with stresses” [83], of which constitutive components are loss of MiL, hopelessness or disheartenment, helplessness, sense of failure, and dysphoria [84,85]. These two constructs are intimately and opposingly connected and have been investigated in our institutions among suicidal individuals who were confronted with extreme life situations that have led to an often-incomprehensible fracture between a “before” and an “after” living experience [84,85,86,87,88,89,90,91,92,93]. In the context of CTE in contact sports, retired athletes experience profound changes in their lives, worsening the outlook and the quality of their living condition. These factors may include reduced autonomy and change in social status, marked by the loss of the above-mentioned “sportive celebrity” condition of both players, which resulted from a training accident before a crucial match and retirement by age restriction.

The majority of the studies have suggested that demoralization is distinguishable from depression [84,85,92]. However, in patients with cancer [94,95,96,97,98,99,100], Parkinson’s disease [101], as well as other psychiatric and medical conditions [89,102,103,104,105], there may be frequent overlaps between demoralization and depression. Nevertheless, demoralization can occur independently of depression and the two conditions are not necessarily linked by a hierarchical connection [89,99,105,106]. In some cases, it has been demonstrated that demoralization had a predominant [89,97,98] or synergistic [99,100,107] effect with depression on the potentiation of SI and SB. In both cases described above, a depressive condition was suspected, and a psychopharmacological treatment was initiated. However, the advanced cognitive decline and the concomitant constellation of psychiatric symptoms made this diagnosis difficult because the latter could mask or modify the expression of cardinal signs and symptoms of major depression. In addition to its connection with depression, the relationship between demoralization and hopelessness has also been documented but remains a debatable subject. In this regard, hopelessness was proposed to only represent a dimension of demoralization rather than the construct itself [84,85,96,102,104,108]. However, this definition of demoralization which contains hopelessness has been questioned for its predictive value over the construct of hopelessness alone [109]. This is particularly relevant for the evaluation of the role of demoralization in suicidality, given the well-established relevance of hopelessness in this domain [78]. The possible modulatory role of hopelessness on how demoralization can lead to suicidality has also been the subject of recent studies [110].

Another possible factor that might potentiate the risk for CTE-associated suicidality in contact sports athletes is the habituation to pain, especially for the severe forms of SB. This process might be deeply intertwined with the training and matches history of contact sportsmen, other athletes, and military personnel [111]. Subsequently, this habituation to pain, while allowing athletes and soldiers to perform well, may have significant drawbacks in the long run. In this regard, the innate perception and response to pain of athletes may be more blunted compared to the general population, who are not consistently exposed to pain and external stressors. Notably, this pathway of pain tolerance has been implicated in the Interpersonal Theory of Suicide [112,113]. This model posits that SI and the desire to die by suicide are fostered by the overlapping of two constructs, the perceived burdensomeness (PB) (the belief that one is a burden to others) and thwarted belongingness (TB) (the belief that one does not belong to a group). The third construct, the acquired capability for suicide (AC), is conceived as an essential prerequisite for executing SI and the desire to die and moving onto SB [112,113,114,115,116,117]. Notably, the AC is reciprocally reinforced by the habituation to the pain and they are both involved in severe forms of SB [112,113,114,115,116,117], as illustrated in reported cases. 

## 4. Limitations

This work has relevant limitations. The failure to perform histological post-mortem examination and the impossibility of executing functional neuroimaging examinations to deepen the differential diagnosis (because of not-consent of patients and their families), make the diagnosis of CTE less consistent. It is also worth emphasizing that while CTE might represent a risk factor for the development of cognitive and mental problems in retired athletes [118], there have been no definitive associations between CTE in this particular CTE patient population and neurodegenerative disease of any types due to the lack of large-scale clinical and epidemiological studies of this pathology [119,120]. Therefore, etiological interpretations of these cases should be taken with caution and in consideration of other co-existing illnesses as well as physical and mental conditions of the patients [120].

## 5. Conclusions

In spite of these important limitations regarding differential diagnosis of CTE, the progressive evolution of traumatic brain injury into chronic neuropsychiatric illness in these case reports exemplify the frequent complex intersection of neurologic and psychiatric disease [45,46]. Howevere, above all, the findings described here would provide clinical pictures for sports physician, neurologist, psychiatrists, and health professionals to improve the identification of patients with CTE and also potential mechanistic insights to refine the knowledge of eventual severe SB development, which might enable its earlier prevention. As predictive medicine is of paramount importance in mental health management [121,122], it appears as imperative that medical personnel be trained to detect possible SI or SB in patients with CTE, particularly in retired athletes. The allocation of additional healthcare resources could be designed to prevent or to timely respond to the possible severe suicidality escalation in these high-risk subjects.

## Figures and Tables

**Table 1:** Comparative neurocognitive assessments of the two reported cases.

Neurocognitive Function	Case 1 October 2007	Case 1 August 2008	Case 2 November 2007
Temporo-spatial orientation	+	−	− −
Immediate memory	+	+	−
Episodic memory encoding Episodic memory retrieval	+ − − −	− − −	− − − −
Naming, oral expression Written language Comprehension	+ + +	+ − −	− + +
Verbal fluency Motor programming Mental flexibility, planning Inhibition	− − − − − −	− − − − − − −	− − − − − − − −
Reasoning	+ −	−	−
Ideomotor gestures Reflexive gestures	+ −	− +	+ +
Visuo-spatial abilities	+	+ −	−
Visual recognition, identification	+	+	+
Anosognosia	+ −	+ −	− −

+, preserved; + −, very mild impairment; −, mild impairment; − −, severe impairment (see text for details).

## Data Availability

Data can be shared after requesting.
